# New York Academy of Medicine

**Published:** 1879-06

**Authors:** 


### NEW YORK ACADEMY OF MEDICINE.

STATED MEETING, May 1, 1879.

Fordyce Barker, M.D., LL.D., President, in the Chair.

After the reading and approving of the minutes, Dr. E. H. Janes, from the Committee on Admissions, reported favorably on the names of the following candidates for resident fellowship, who were afterward duly elected: E. Sanders, Glover C. Arnold, John J. Milhau, Clement Cleveland, William H. Welch, John Shrady and F. P. Chambers.

There were present, as guests, Dr. David King, of Newport, and Dr. J. W. Roseburgh, of Hamilton, Canada; and these gentlemen were invited by the President to take seats beside him.

The Librarian, Dr. Laurence Johnson, announced that since his last report there had been received thirty-eight bound volumes, seventeen unbound volumes, and eight-hundred and seventy-seven pamphlets and journals, and mentioned a number of the names of the donors.

The Corresponding Secretary, Dr. John G. Adams, then made a brief report, and also presented to the Academy a framed copy of the programme of the old Broome Street School of Medicine, which was organized in 1836. It was started by six physicians and surgeons, of whom Dr. Post and himself were now the only survivors, and he believed that in it the first course of spring and autumn lectures ever given in New York was delivered. It was also, as far as he knew, the first private medical school ever gotten up in this city.

After a report by the Committee of Ways and Means, through its Chairman, Dr. James Anderson, the paper of the evening was read by Frank H. Hamilton, M.D., LL.D., on "Posture as a means of relief in strangulated and incarcerated hernia, with a general consideration of the mechanism of reduction."

In a late number of the *British Medical Journal* there had been published an interesting account of a case of intussusception in a child, which was cured by means of copious injections of thin gruel into the bowel. Dr. Blaker, the physician in whose practice it occurred, attributed the good result obtained mainly to the three following points:

1. Complete anæsthesia, induced by chloroform.
2. An early resort to the injection of gruel.
3. The position of the child when the injections were made, viz.: lying on its back, with the nates raised upon a pillow.

Dr. Hamilton was inclined to attribute more benefit to the last agency than the gentleman reporting the case seemed to do, and the main object which he had in writing the present paper was to set forth his views in regard to the importance of posture in the reduction of strangulated hernia, as well as the reasons why certain positions of the body were of service. When he first contemplated it, he intended it to include a consideration of spasmodic colic and illius also; but, on account of the extensiveness of the subject of hernia, he was obliged to postpone those topics until some other occasion.

He had collected fourteen cases of strangulated hernia occurring in his own practice, in which a reduction had been accomplished mainly through the agency of posture, but for lack of time he would be able to recite only two or three of them as specimens.

Case No. 3 of these was as follows: In 1865, while on duty as one of the attending surgeons to Charity Hospital, his attention was called by the house-surgeon to a male convict suffering from an indirect inguinal hernia which had become strangulated. When he first saw him ineffectual efforts at reduction had been kept up during the whole of the preceding night, and the man was now very anxious to take an emetic, as he said that once before, when his hernia had become strangulated, this had relieved him. It was deemed unadvisable to administer an emetic; but while the house-surgeon was making the preparations for an operation, which it was feared would have to be resorted to, Dr. Hamilton concluded to try the effect of placing the man on a steep inclined plane, with the head downward. Accordingly, the foot of the bed was elevated until its legs rested upon a table; when, in about ten minutes, he had the satisfaction of seeing the hernial tumor disappear altogether.

Another case was related very similar to this, which had occurred previously in Buffalo. In 1871 Dr. Hamilton was called to see, in consultation, a gentleman who three days before had been seized with a severe pain in the region of the gall-bladder, which was at first naturally supposed to be due to the passage of a gall-stone. Somewhat more than twenty-four hours before he was called in, however, it was discovered that a hernia (although he had never before had anything of the kind) had descended into the scrotum, and was now strangulated.

He directed that the feet of the patient should be lifted on the shoulders of a strong man; but, after this position had been tried for some time, the strangulation still continued as before. Complete anæsthesia by means of ether was now secured, and then the experiment of elevating the feet in the same manner was again tried, when immediately there followed a successful reduction.

Besides the fourteen cases mentioned above, Dr. Hamilton had seen quite a large number of others in which reduction had been secured by means of posture, but of these he had preserved no special record. Then, in former papers published by him in the Bellevue and Charity Hospital reports, notes had been given of twenty-three cases in which other methods in addition to that of posture had been employed; but even in these he regarded this as the chief agency of successful reduction.

In order that the mechanism of reduction might be properly appreciated, it was necessary that we should arrive at some definite conclusion as to the true causes of strangulation. It had long been taught by various authorities that muscular spasm was a most important element in its causation, and in support of this view the writer quoted a passage from Velpeau, which concluded as follows: "All muscular contraction *must* increase the strangulation." Sir Astley Cooper, in speaking of strangulation at the upper ring, stated that a portion of the intestine protruded under the transversalis and internal oblique muscles, and was compressed by them. Ferguson spoke of the use of anti-spasmodics in reduction, and many other authors might have been quoted to the same effect, if time had permitted.

Dewitt said that muscular spasms was *formerly* considered to be a cause of strangulated hernia; but the writer feared that many surgeons still retained this old idea. Dr. Hamilton believed that Velpeau and Sir Astley Cooper were entirely wrong in their opinions on this subject, and he remarked that whatever anatomical reason might be cited in favor of their view, there remained a sufficient pathological reason why this was erroneous, viz.: that muscular spasm was always too intermittent and brief in duration to constitute the cause of a condition like strangulated hernia. Such a view of the causation could not, therefore, be accepted, notwithstanding the high authorities that could be quoted in its support. Skey not only positively denied the agency of muscular spasm, but stated that this was now altogether an antiquated and exploded notion. Antiquated it undoubtedly was; but he regretted that it was hardly as yet to be considered as exploded, since so many still seemed to adhere to it, in spite of the arguments that were adduced to disprove it.

The next point to investigate was, what was the effect of normal muscular tension. As a general rule, the position of the patient had no effect upon hernial apertures. Flexing and rotating the thigh inward was frequently recommended in order to facilitate the return of a hernia; but it could be easily shown that such manœuvres were in no way instrumental in relaxing the internal ring, at which the strangulation exists in the vast majority of cases. In hernias of long standing the canal usually acquired an almost cartilaginous hardness, and it was not, therefore, capable of being either enlarged or diminished by posture. Consequently, the diameter of the ring was not affected; and besides, in a considerable proportion of cases the strangulation occurred in the sac itself in these old hernias. The conclusion that must be arrived at, then, was that there were but very few cases in which either muscular spasm or normal muscular action were capable of influencing hernial apertures. In femoral hernia the internal or crural ring was almost always the seat of stricture, and it had long ago been demonstrated that posture had no effect in giving relief here. Velpeau and other authorities had acknowledged this; and one reason was the important anatomical position that Gimbernat's ligament held in reference to this condition.

We had now to consider whether in diaphragmatic and ventral hernias (where there were no natural openings), muscular spasm might not be directly concerned in the causation. In consequence of its position, diaphragmatic hernia was entirely removed from observation; but the writer had had the opportunity of seeing a large number of cases of ventral hernia produced by stabs and other similar wounds, as well as some due to gunshot injuries, which one would naturally suppose to be a more frequent cause of this than is actually the case. All such strangulated hernias were difficult to reduce, until the patient had been brought under the influence of an anæsthetic. But in no case had simple relaxation of the muscles proved sufficient; and this was not difficult to explain. In all strangulated hernias the hernial opening was stretched to the utmost. If it were not, there would of course be no strangulation. Consequently, instead of a simple slit, there was now a circular aperture. If we were to suppose, for example, that there was a body like a finger or a piece of omentum blocking up such an opening, it would be silly to imagine that any amount of muscular relaxation would release it. The same was true in regard to the intestine, which, as a general rule, practically represented a solid mass.

By what other means, whether local or general, could we hope to relax these apertures? The old surgeons were accustomed to resort to such local measures as the application of warm fomentations, liniments, belladonna ointment, etc. Even at the present day there were good surgeons who recommended warm fomentations, and Prof.

Gross advocated cold fomentations; but he believed it would be a difficult task for such authorities to show how fomentations, whether warm or cold, could have the effect of relaxing such openings. There was much more speciousness in the idea that this result could be accomplished by the employment of such general agents as chloroform or other anæsthetics, blood-letting, tobacco-enemata, etc.

That such means were instrumental in relaxing the apertures had seemed a necessary inference; but Dr. Hamilton was not willing to accept this conclusion, since he did not believe that it was founded on sound anatomical and pathological premises. On the other hand, there were more satisfactory explanations of which we might avail ourselves; and it was important to bear in mind in this connection that it was the muscular fibres alone (and not the tendons of muscles), which were relaxed by such agents.

What, then, was the true theory of reduction in the great majority of instances? It had been sufficiently demonstrated, he thought, that it did not ordinarily consist in the relaxation of the hernial canal or rings; and he would now endeavor to show that the reduction was in reality effected by the employment of external pressure, in the form of taxis, and by internal traction—the operation of these forces always being greatly facilitated by paralysis of the muscles. Of the value of taxis, tested as it had been by such long and universal experience, there could be no doubt; but of the practical efficiency of internal traction less was understood by the profession. This kind of traction might be caused in a variety of ways. Thus, emetics might produce it by the sudden upheaval of the abdominal viscera toward the upper portion of the cavity which they occasioned; and the same was true of cold water dashed suddenly upon the bare skin. In both cases, however, there was also a contraction of the muscles. Emetics, in addition, had sometimes the effect of inducing a violent anti-peristalsis in the intestines. Cathartics were probably of service only by exciting peristaltic or anti-peristaltic movements, and Skey had stated that they caused a dragging-up of the bowel from the sac. But inactual practice neither emetics nor cathartics were often now employed; because, if they were not successful in causing a reduction, they were apt to do harm by increasing the tendency to inflammatory action. In order to facilitate whatever means were made use of, it was generally recommended that the bladder should be emptied, and that, if there was any accumulation of gas in the lower part of the bowels, it should be removed. For the latter purpose, as well as the removal of fæcal matter, enemata were serviceable, and, besides, sometimes had the effect of exciting violent peristaltic efforts. Tobacco-enemata had long been held in repute in strangulated hernia, and were of great service in relaxing the abdominal muscles; but Dr. Hamilton could not believe that this or any other agent (except, perhaps, in very rare instances) was capable of causing an enlargement of the hernial openings. Still, even when tobacco was employed, he thought that the peristalsis set up by it was its most efficient result, and that the muscular relaxation resulting from its use was of secondary importance to this. It was an agent not without danger, however, and death had been known to follow from the prostration which it caused. Chloroform, bleeding and warm baths all had the effect of relaxing the muscles which resisted the return of the hernia (not of relaxing the apertures), and of these chloroform was the most efficient. Of how much service such an effect was, we could better appreciate if we considered the subject of abdominal wounds for a moment. In knife-stabs and other short wounds in the abdomen, it was almost impossible to prevent extrusion of the viscera; while in large incisions, such as are made in ovariotomy, for instance, there was no tendency of this kind, because the great muscles were thereby completely relaxed. In the same manner the muscles were paralyzed by such agents as those mentioned above, and, consequently, their resistance to taxis overcome. The effect of opium was probably much the same, although its action was somewhat more difficult to explain.

Dr. Harrison at this point wished to make the suggestion that while the patient was under the influence of such remedies, and the abdominal muscles thus relaxed by them, the hernia might actually be withdrawn by violent peristalsis or anti-peristalsis in the intestine. It was well known that peristaltic movements often continued for some time after death, and he himself had seen this demonstrated in several instances in the case of calves. Niemeyer and other observers had noticed a number of invaginations of the intestines at the autopsies of children who had died of hydrocephalus, which had been produced by the violence of *post-mortem* peristaltic movements. Might it not be true that in the same way these peristaltic movements went on under the temporary paralysis produced by chloroform, bleeding, etc., just as in labor uterine contractions continued, and were sometimes increased when the patient was under the influence of an anæsthetic? It seemed reasonable to suppose, therefore, that one way, at least, in which the above agents acted was by putting the abdominal muscles in such a condition that their contractions could not interfere with the continuance or increase of peristalsis.—*Medical Record.*

